# Strain features of pneumococcal isolates in the pre- and post-PCV10 era in Pakistan

**DOI:** 10.1099/mgen.0.001163

**Published:** 2024-01-25

**Authors:** Nida Javaid, Stephanie W. Lo, Muhammad Imran Nisar, Asma Basharat, Hadiqa Jaleel, Karam Rasool, Qamar Sultana, Furqan Kabir, Aneeta Hotwani, Robert F. Breiman, Stephen D. Bentley, Sadia Shakoor, Shaper Mirza

**Affiliations:** ^1^​ Department of Life Sciences, School of Science and Engineering, Lahore University of Management Science, Lahore, Pakistan; ^2^​ Parasites and Microbes, Wellcome Sanger Institute, Hinxton, UK; ^3^​ Milner Centre for Evolution, Department of Life Science, University of Bath, Bath, UK; ^4^​ Departments of Pathology, Pediatrics, and Medicine, Aga Khan University, Karachi, Pakistan; ^5^​ Department of Microbiology, Chughtai Lab/Chughtai Institute of Pathology, Lahore, Pakistan; ^6^​ Infectious Diseases Research Laboratory (IDRL), Dept. of Paediatrics & Child Health, Aga Khan University, Karachi, Pakistan; ^7^​ Department of Global Health, Rollins School of Public Health, Emory University, Atlanta, GA, USA

**Keywords:** antimicrobial resistance, pneumococcal conjugate vaccine, pneumococcal genomics

## Abstract

Pakistan is amongst the four countries with the highest number of pneumococcal deaths. While the PCV10 vaccine was introduced in Pakistan in October 2012, data regarding the impact of the vaccine on the population dynamics of *Streptococcus pneumoniae* in Pakistan remain obscure. Using whole genome sequencing of 190 isolates (nasopharyngeal carriage=75, disease=113, unknown sites=2) collected between 2002 and 2020, this study presents characteristics of pneumococcal strains in Pakistan in the pre- and post-vaccine era. The isolates were characterized on the basis of serotype distribution, genetic lineages (or Global Pneumococcal Sequence Cluster, GPSC) and antibiotic resistance. A high level of diversity in serotype and genetic lineages of pneumococci was observed in Pakistan. Among 190 isolates, we identified 54 serotypes, 67 GPSCs and 116 sequence types (STs) including 23 new STs. The most prevalent GPSCs and their associated serotypes in nasopharyngeal carriage were GPSC54 (expressing serotype 9V), GPSC5 (15A and 7B, and serogroup 24), GPSC25 (15B/15C), GPSC67 (18C) and GPSC376 (6A and 6D). Similarly, among 113 disease-causing isolates, the most prevalent GPSC/serotype combinations were GPSC2 (serotype 1), GPSC10 (serotypes 14, 10A, 19A and 19F), GPSC43 (serotypes 13, 11A, 23B, 35A and 9V), GPSC67 (serotypes 18A and 18C) and GPSC642 (serotype 11A). Of the 190 isolates, the highest levels of resistance were observed against penicillin (58.9 %, *n*=122), erythromycin (29.5 %, *n*=56), clindamycin (13.2 %, *n*=25), co-trimoxazole (94.2 %, *n*=179) and tetracycline/doxycycline (53.2 %, *n*=101). A higher proportion of disease-causing isolates were multidrug resistant as compared to carriage isolates (54 % vs 25 %). Our data suggest limited coverage of PCV10 in nasopharyngeal (21.6 %, 16/74) as well as disease-causing (38.1 %, 16/42) isolates among children ≤5 years old; however, higher valent vaccine PCV13 would increase the coverage rates to 33.8 % in nasopharyngeal and 54.8 % in disease-causing isolates, whereas PCV24/25 would offer the highest coverage rates. Owing to the diversity of serotypes observed during the post-vaccine period, the suggested inclusion of serotype in future vaccine formulations will require investigations with larger data sets with an extended temporal window. This article contains data hosted by Microreact.

## Abbreviations

AMR, antimicrobial resistance; CDC, center for disease control and prevention; CLSI, Clinical and Laboratory Standards Institute; DDD, defined daily dosage; EPI, expanded program on immunization; GPSC, global pneumococcal sequence cluster; MDR, multi-drug resistance; MIC, minimum inhibitory concentration; NVT, non-vaccine type; PCV, pneumococcal conjugate vaccine; ST, sequence type.

## Impact Statement

Using pneumococcal strains from both the pre- and post-vaccine era, we were able to characterize pneumococcal isolates in Pakistan over a 15 year period. The study provides an understanding of serotypes that are commonly associated with disease and carriage in Pakistan, and provides data on the genotypes associated with the vaccine serotypes. These findings describes the circulating serotypes in the pre- and post-PCV10 era in Pakistan. This, in turn, would may assist policy -makers to in makeing decisions regarding continuation of PCV13 which was recently been introduced in Pakistan in 2021, improvements in PCV10/PCV13 coverage or use of higher valency vaccines as a booster in children previously vaccinated with PCV10/13.

## Data Summary

Whole-genome sequences are deposited at the European Nucleotide Archive (ENA) and accession numbers are available with the complete metadata of this study (Table S1, available in the online version of this article). A phylogenetic snapshot of pneumococcal isolates from Pakistan is available at https://microreact.org/project/qYqCAfqPSgwyPASKUZ6UFf/d98a9ce5. The authors confirm that all supporting data, code and protocols have been provided within the article or through supplementary data files.

## Introduction

*Streptococcus pneumoniae* remains an important cause of pneumonia in children and the elderly. In addition to pneumonia, it can also cause an array of infections, ranging from mild localized otitis media, to more severe pneumonia, sepsis and meningitis [1]. In 2015, pneumococcal infections killed an estimated 317 000 children under 5 years of age worldwide [2]. A significant proportion of these infections occurred in developing countries including Pakistan. With a total of 14 400 deaths (with an uncertainty range of 9700–17000), Pakistan is ranked fourth in the global burden of pneumococcal disease [2].

To reduce the burden of pneumococcal infection, Pakistan was the first among South Asian countries to introduce the 10-valent pneumococcal conjugate vaccine (PCV10) in its routine immunization programme in October 2012. The regimen currently in use in Pakistan is three doses given at 6, 10 and 14 weeks of life with no catch-up programme [3]. Between 2013 and 2015, PCV10 coverage was reportedly increasing (65 % in 2013 to 80 % in 2015), but it plateaued in 2016. According to the latest World Health Organization update, PCV10 uptake was 83 % in 2019 [4]. Recently, PCV13 was introduced in the Expanded Program on Immunization (EPI) of Pakistan in 2021; investigations need to be carried out on the impact of PCV13 on pneumococcal population dynamics.

Despite high mortality rates associated with pneumococcal infections and recommendations for continuous surveillance of the pneumococcal population at the regional level, only a limited number of studies have reported on serotype distribution of pneumococci in Pakistan. Previous studies have reported high serotype diversity and low coverage (30–74 %) of circulating serotypes by the currently available PCVs (PCV10 and PCV13) [3, 5–10]. However, none of these studies evaluated the diversity of pneumococcal lineages [i.e. Global Pneumococcal Sequence Clusters (GPSCs)] and their association with major serotypes as shown for other regions [11, 12].

The current study therefore aimed to investigate the distribution of serotypes, and to relate this to the pneumococcal lineages and resistance profiles of pneumococcal isolates collected from carriage and disease-causing cases in Pakistan between 2006 and 2020 using whole-genome sequencing.

## Methodology

### Study design

Ethical approval of the study was given by the Institution Review Boards of Children Hospital, Lahore University of Management Sciences (LUMS) and Aga Khan University (AKU).

For the collection of nasal swabs from children, the study was explained to mothers (mostly children are accompanied by their mothers), and consenting mothers were requested to sign (or use a thumb print if not able to sign the form) the consent form. Once the form was signed, nasopharyngeal samples were collected by trained pathology staff.

A total of 190 *S*. *pneumoniae* isolates were collected between 2006 and 2020 primarily from the provinces of Sindh and Punjab, the two major provinces of Pakistan, which make up 76 % (158/208 million) of the total Pakistani population [13]. Ninety-eight isolates were collected at AKU Hospital; of these, 37 pneumococcal isolates were clinical isolates collected and archived at Aga Khan University Hospital as part of routine microbiological culture and antimicrobial susceptibility testing between 2006 and 2016 [9] while the remaining 61 nasopharyngeal isolates were collected during a survey designed to determine pneumococcal serotype distribution in carriage isolates from unvaccinated healthy children with no respiratory symptoms (≤5 years of age) before the introduction of PCV10 in Sindh [7]. Ninety-two isolates were collected at LUMS. Of these, 13 nasopharyngeal isolates were collected from Punjab during 2019–2020 from vaccinated children under the age of 6 years reporting to the paediatric clinic at The Children Hospital, Lahore, for infections other than upper respiratory tract infection, 76 were disease-causing isolates, two isolates lacked information on site of isolation, and one nasopharyngeal (from nasal secretion) isolate was collected between 2016 and 2020 from a private clinical laboratory (Chughtai Lab Lahore), where samples were submitted for routine culture and antimicrobial susceptibility testing. Phenotypic resistance profiles were submitted with the isolates and were determined using Clinical and Laboratory Standards Institute (CLSI) guidelines for disc diffusion and microbroth dilution methods [14]. In summary, 113 disease-causing isolates, 14 nasopharyngeal isolates from patients with non-upper respiratory infections, 61 nasopharyngeal isolates from healthy children and two isolates from an unknown site of isolation were included. In downstream analysis, the two isolates from an unknown site of isolation were included in the total isolates but were not grouped into nasopharyngeal or disease-causing isolates. We grouped 75 isolates into nasopharyngeal isolates and 113 into disease-causing isolates (from both invasive and non-invasive sites of isolation), and their respective demographic data are shown in Table 1.

**Table 1.** Demographics of the pneumococcal collection from nasopharynx swabs (*n*=75) and pneumococcal disease-causing (*n*=113) isolates, Pakistan, 2006–2020

	Nasopharyngeal swabs	Disease-causing	Total
**Gender**			
Female	42.7 % (32/75)	41.6 % (47/113)	42.0 % (79)
Male	57.3 % (43/75)	58.4 % (66/113)	58.0 % (109)
**Vaccine period**			
Pre-PCV10	81.3 % (61/75)	16.8 % (19/113)	42.6 % (80)
Post-PCV10	18.7 % (14/75)	83.2 % (94/113)	57.4 % (108)
Age (years)*			
≤5	98.7 % (74/75)	37.5 % (42/112)	62.0 % (116)
>5	1.3 % (1/75)	62.5 % (70/112)	38.0 % (71)
**Province**			
Sindh	81.3 % (61/75)	33.6 % (38/113)	52.7 % (99)
Punjab	18.7 % (14/75)	54.0 % (61/113)	39.9 % (75)
Khyber Pakhtunkhwa	0	9.7 % (11/113)	5.9 % (11)
Azad Jammu and Kashmir	0	2.7 % (3/113)	1.6 % (3)
**Year**			
2006	0	7.1 % (8/113)	4.3 % (8)
2007	0	1.8 % (2/113)	1.1 % (2)
2009	0	0.9 % (1/113)	0.5 % (1)
2010	0	2.7 % (3/113)	1.6 % (3)
2011	0	0.9 % (1/113)	0.5 % (1)
2012	0	0.9 % (1/113)	0.5 % (1)
2013	81.3 % (61/75)	2.7 % (3/113)	34.0 % (64)
2014	0	5.3 % (6/113)	3.2 % (6)
2015	0	7.1 % (8/113)	4.3 % (8)
2016	1.3 % (1/75)	19.5 % (22/113)	12.2 % (23)
2017	0	19.5 % (22/113)	11.7 % (22)
2018	0	20.4 % (23/113)	12.2 % (23)
2019	10.7 % (8/75)	9.7 % (11/113)	10.1 % (19)
2020	6.7 % (5/75)	1.8 % (2/113)	3.7 % (7)

For two isolates, information on their site of isolation, age, gender and city of sample collection was not available. These two isolates are not included in the table.

*For one isolate, information on age was missing and it was not included in the age section.

### Pneumococcal strain confirmation

Presumptive pneumococcal isolates were grown on 5 % sheep blood-agar plates (Hardy Diagnostics) at 37 °C in 5 % CO_2_ overnight. The purity of each culture and species confirmation were determined by conventional methods including, Gram staining (Sigma), alpha haemolysis and optochin sensitivity (Becton, Dickinson and Company). Purified strains were kept frozen at −80 °C until later use.

### DNA extraction and serotyping using sequential multiplex PCR

Extraction of pneumococcal DNA was performed by growing frozen bacterial stocks (stored at −80 °C) on blood agar plates and using the DNA extraction protocol described by the Centers for Disease Control and Prevention (CDC) (https://www.cdc.gov/streplab/downloads/pcr-dna-extraction-culture-may2009.pdf). Briefly, a bacterial pellet was washed with Tris-EDTA buffer (pH 8.0) and lysed by incubating at 65 °C in the presence of 10 % SDS (Sigma Aldrich) and 2 µl of 10 µg ml^−1^ lysozyme for 15 min, followed by addition of 70 µl of 5 M potassium acetate. After 10 min of incubation at 65 °C, phases were separated by spinning at 12 000 r.p.m. for 10 min. The top layer containing DNA was placed in a clean Eppendorf and was precipitated using 95 % cold ethanol. DNA was pelleted by spinning at 12 000 r.p.m. for 5 min and the pellet was resuspended in sterile DNase-free water to adjust the concentration to 50 ng µl^–1^ and stored at −20 °C. The extracted DNA was also used to serotype the isolates using a conventional multiplex PCR protocol for *S. pneumoniae* serotyping (CDC) (https://www.cdc.gov/streplab/pneumococcus/resources.html). The multiplex PCR serotyping results were then compared with serotypes inferred from the genomic data to investigate the concordance.

### Genomic sequencing and analysis

Pneumococcal DNA was prepared and genomes were sequenced on an Illumina HiSeq platform which produced paired-end sequence reads of 151 bp in length, and quality control on sequence reads and assemblies were performed as previously described for the global pneumococcal dataset [11]. Serotypes and sequence types (STs) were inferred from the genome data using SeroBA [15] and GetST using the pubMLST database [16]. Antibiotic resistance profiles of 17 antibiotics, including penicillin, amoxicillin, meropenem, cefotaxime, ceftriaxone, cefuroxime, erythromycin, clindamycin, quinupristin/dalfopristin, linezolid, cotrimoxazole, tetracycline, doxycycline, levofloxacin, chloramphenicol rifampin and vancomycin, were predicted from the genomic data using the CDC AMR pipeline [17–19]. Minimum inhibitory concentrations (MICs) were interpreted using CLSI guidelines [14] to categorize the isolates into resistant, intermediate and susceptible. According to non-meningitis cutoffs, all isolates were susceptible to penicillin, cefotaxime and ceftriaxone [14]. The more stringent meningitis cutoffs were used for interpreting MICs against penicillin, cefotaxime and ceftriaxone throughout the study so as to increase our ability to capture non-susceptible strains [14]. For analysis, isolates that tested as intermediate and fully resistant were both grouped as resistant. Multidrug resistance (MDR) was defined as resistance to three or more classes of antibiotics. To compare isolates from Pakistan with isolates in the Global Pneumococcal Sequencing (GPS) project database, each genome was assigned to a GPSC using Population Partitioning Using Nucleotide K-mers (PopPUNK) [20]. The GPSC reference database used to designate the GPSCs to each strain was downloaded from the GPS website (https://www.pneumogen.net/gps/assigningGPSCs.html). A maximum likelihood tree was reconstructed based on SNPs by mapping to a reference genome of *S. pneumoniae* ATCC 700669 (NCBI accession number FM211187) using FastTree [21]. We used ATCC 700669 (Spain^23F^-1, PMEN1) as a reference genome because genomes generated from the GPS project ran through a standard pipeline in which mapping was carried out using PMEN1 as reference. An interactive visualization of the phylogenetic tree and the metadata was created using Microreact (https://microreact.org/project/qYqCAfqPSgwyPASKUZ6UFf/77268fff) [22].

### Detection of capsular switching

To detect potential capsular switching events in Pakistan, we identified STs expressing more than one serotype within the Pakistan dataset [23]. Next, GPSCs for these STs were selected and the metadata for the global isolates belonging to these GPSCs were downloaded from the GPS project website (https://www.pneumogen.net/gps/GPSC_lineages.html; last accessed on 1 September 2021). Next, we compared the serotypes within each GPSC in Pakistan with the global strains. The serotype–ST combinations not detected in the global databases provided on GPS and pubMLST (https://pubmlst.org/bigsdb?db=pubmlst_spneumoniae_isolates&page=profiles; last accessed on 1 September 2021) were selected for downstream analysis, in which genetic relatedness of isolates from Pakistan was determined by constructing a lineage-specific tree using the global collection of genomes belonging to the lineage of interest.

To construct a lineage-specific tree for each GPSC of interest, reads were assembled to a lineage-specific reference genome using the Burrrows–Wheeler Aligner [24] and Gubbins was used to remove recombination sites, and create a recombination-free alignment [25]. We constructed lineage-specific trees with the isolates from different regions to understand the relatedness of Pakistan isolates with the global isolates and infer: (1) whether the capsular switching event occurred in Pakistan (when the isolates from Pakistan cluster together on the tree) or (2) whether an importation event occurred where the strain may have been imported from another country to Pakistan (when the isolates from Pakistan cluster with the isolates from other countries).

### Statistical analysis

Isolates were grouped as follows based on the period during which they were collected: (1) pre-PCV10 era – samples collected before the introduction of PCV10 between 2006 and 2013, and (2) post-PCV10 era – isolates collected after the introduction of PCV10 between 2013 and 2020. Results for predicted *in silico* serotypes were compared with the results obtained from multiplex PCR for serotyping using the exact function in MS Excel. *In silico* serotypes were used for downstream analysis. Serotypes were grouped into two categories: those covered by PCV10 (PCV10 serotypes: 1, 4, 5, 6B, 7F, 9V, 14, 18C, 19F and 23F), and serotypes not covered by PCV10 [non-vaccine types (NVTs)]. Further, serotypes 15B and 15C were grouped together into 15B/15C because their interconversion rate is very high [26]. For age group comparisons, we stratified disease-causing isolates into (1) ≤5 years and (2) >5 years of age.

Differences in the distribution of GPSCs, serotypes and resistance to each antibiotic over age, gender and site of isolation were assessed using Fisher’s Exact Test. Two-sided *P*-values of <0.05 were considered significant. Multiple testing correction was done using the Benjamini–Hochberg false discovery rate of 5 % when the number of tests was >10. Statistical tests were performed in R version 3.5.2.

## Results

### Bacterial collection

Of the 75 nasopharyngeal isolates, 61 (76.2 %) were from pre-PCV10 and 14 (13 %) were from the post-PCV10 era (Table 1). In comparison with nasopharyngeal isolates, a higher proportion of disease-causing isolates (70/112; 62.5 %) were collected from adults as compared to children (42/112; 37.5 %) (Table 1). Further, most disease-associated isolates were from the post-PCV10 period. The distribution of the samples over nasopharyngeal and disease-causing isolates is summarized in Table 1. The highest proportion (31 %) of disease-causing isolates was recovered from blood, followed by sputum (19.5 %) and pus (15 %) (Table S2).

### Distribution of pneumococcal serotypes

**Fig. 1.**
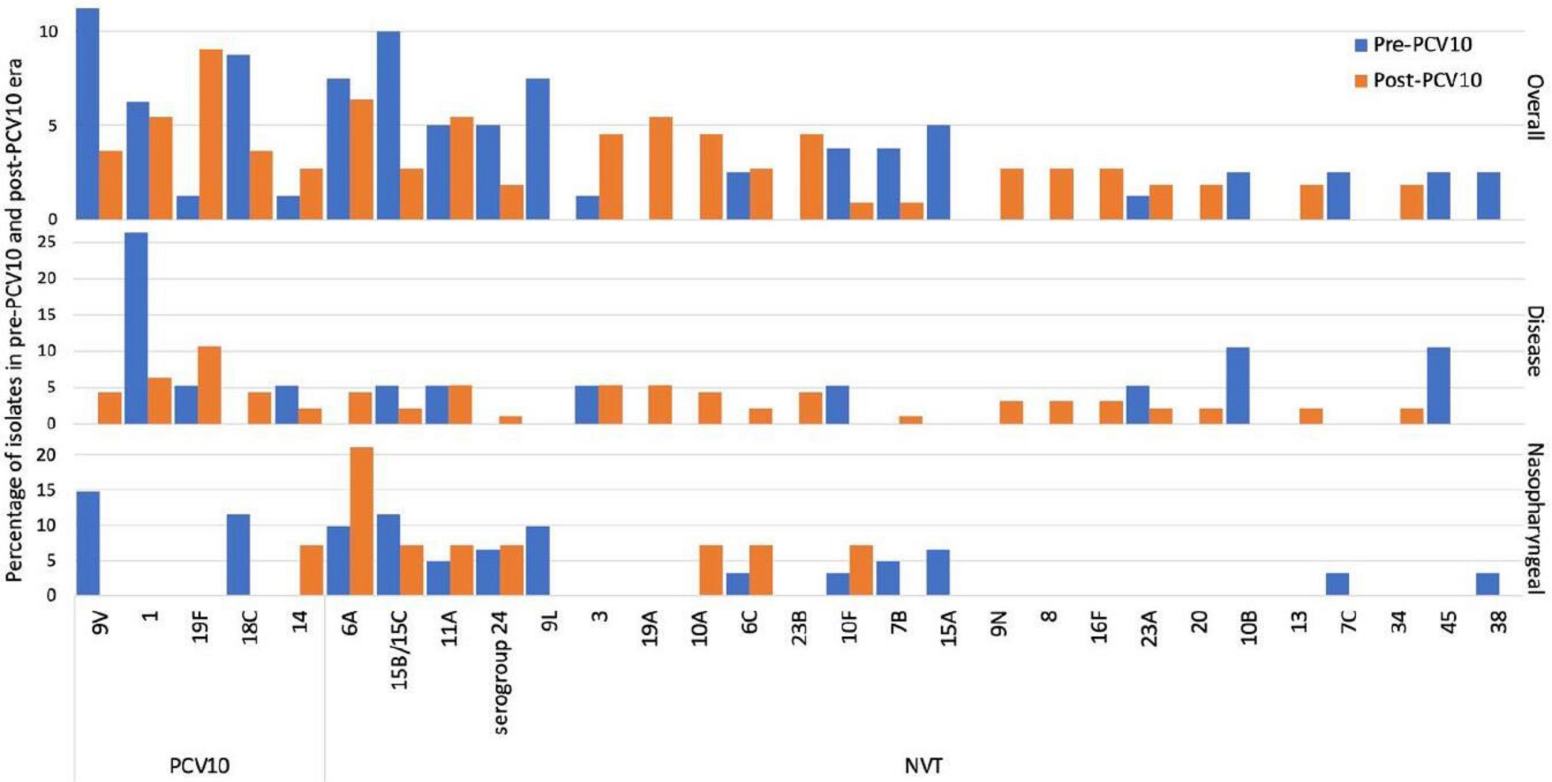
Distribution of pneumococcal serotypes before and after the introduction of PCV10 in Pakistan. Serotypes with at least two isolates in the complete dataset are shown. This figure shows the percentage of isolates of each serotype in the pre-PCV10 (blue) and post-PCV10 era (orange) in the complete dataset, and disease and nasopharyngeal isolates, respectively.

There was a 100 % concordance between predicted *in silico* serotypes and the results obtained from multiplex PCR. Overall, 53 serotypes were detected in 190 pneumococcal isolates. Of these, 21 serotypes were expressed by only a single isolate. The most common serotypes included 9V (*n*=13), 6A (*n*=13), 15B/15C (*n*=11), 18C (*n*=11), 1 (*n*=11) and 19F (*n*=11) (Fig. 1). These common serotypes were covered by PCV10, except for serotypes 6A and 15B/15C. Higher proportions of serotype 1 (*P*=0.048) and 19F (*P*=0.048) were isolated from disease-causing samples whereas serotype 9L (*P*=0.048) was primarily detected in nasopharyngeal isolates (Fig. S1 and Table S3). Further, serotype 2 was not detected in this dataset. No significant association was observed between serotypes and the patients’ demographics (age and gender) as shown in Fig. S1.

The most common serotypes isolated from the 75 nasopharyngeal samples were 9V (*n*=9), 6A (*n*=9), 15B/C (*n*=8), 18C (*n*=7) and 9L (*n*=6), whereas the most common serotypes among the disease-causing isolates were 19F (*n*=11/113), 1 (*n*=11), 11A (*n*=6), 3 (*n*=6) and 19A (*n*=5) (Fig. 1). To determine if pneumococcal vaccines covering a greater number of serotypes would provide better coverage against serotypes causing infection in our population, we compared differences in coverage by pneumococcal vaccines including PCV10, PCV13, PCV15, PCV20, PCV24, PCV25 and Pneumosil [a 10-valent PCV produced by the Serum Institute of India (SII) containing the most prevalent invasive serotypes in India including 1, 5, 6A, 6B, 7F, 9V, 14, 19A, 19F and 23F]. Overall, 35.4 % (40/113) of the disease-causing isolates were included in PCV10 (expressing serotypes covered by PCV10) in comparison to nasopharyngeal isolates, where only 22.7 % (17/75) of isolates were covered by PCV10 (Table 2). The highest proportion of nasopharyngeal isolates was covered (54.7 %) by PCV25 whereas the highest proportion of disease-causing isolates (69 %) was covered by PCV24 (Table 2).

**Table 2.** Current and expected PCV coverage of pneumococcal serotypes from nasopharynx swab (*n*=75) and pneumococcal disease (*n*=113) isolates, Pakistan, 2006–2020; data are *n* (%)

Vaccine	Nasopharyngeal isolates (*n*=75)	Disease-causing isolates (*n*=113)	Disease-causing isolates from children ≤5 years (*n*=42)
PCV10	17 (22.7 %)	40 (35.4 %)	16 (38.1 %)
PCV13	26 (34.7 %)	55 (48.7 %)	23 (54.8 %)
PCV15*	26 (34.7 %)	56 (49.6 %)	23 (54.8 %)
PCV20†	40 (53.3 %)	72 (63.7 %)	27 (64.3 %)
PCV24‡	40 (53.3 %)	78 (69 %)	28 (66.7 %)
PCV25/IVT-25§	41 (54.7 %)	73 (64.6 %)	114 (60.6 %)
Pneumosil¶	19 (25.3)	43 (38.1 %)	20 (69 %)

*PCV15 is under review for use in individuals >18 years old.

†PCV20 is approved by the FDA for use in individuals >18 years old.

‡PCV24 is under development.

§VT-25 is a PCV25 that covers against serotypes responsible for causing invasive infections in children in low- and middle-income countries. As PCV25 contains 24F, serogroup 24 was included in PCV25 types.

¶Pneumosil is a PCV10 that covers against serotypes 6A and 19A.

### Distribution of global pneumococcal sequencing clusters

We detected 66 GPSCs and 113 STs among 190 isolates. Of these 66 GPSCs, 30 were singletons (represented by a single isolate) and 12 had two isolates each (Fig. S3). We observed a close linkage between STs and GPSC, in that all STs were associated with only a single GPSC.

**Fig. 2.**
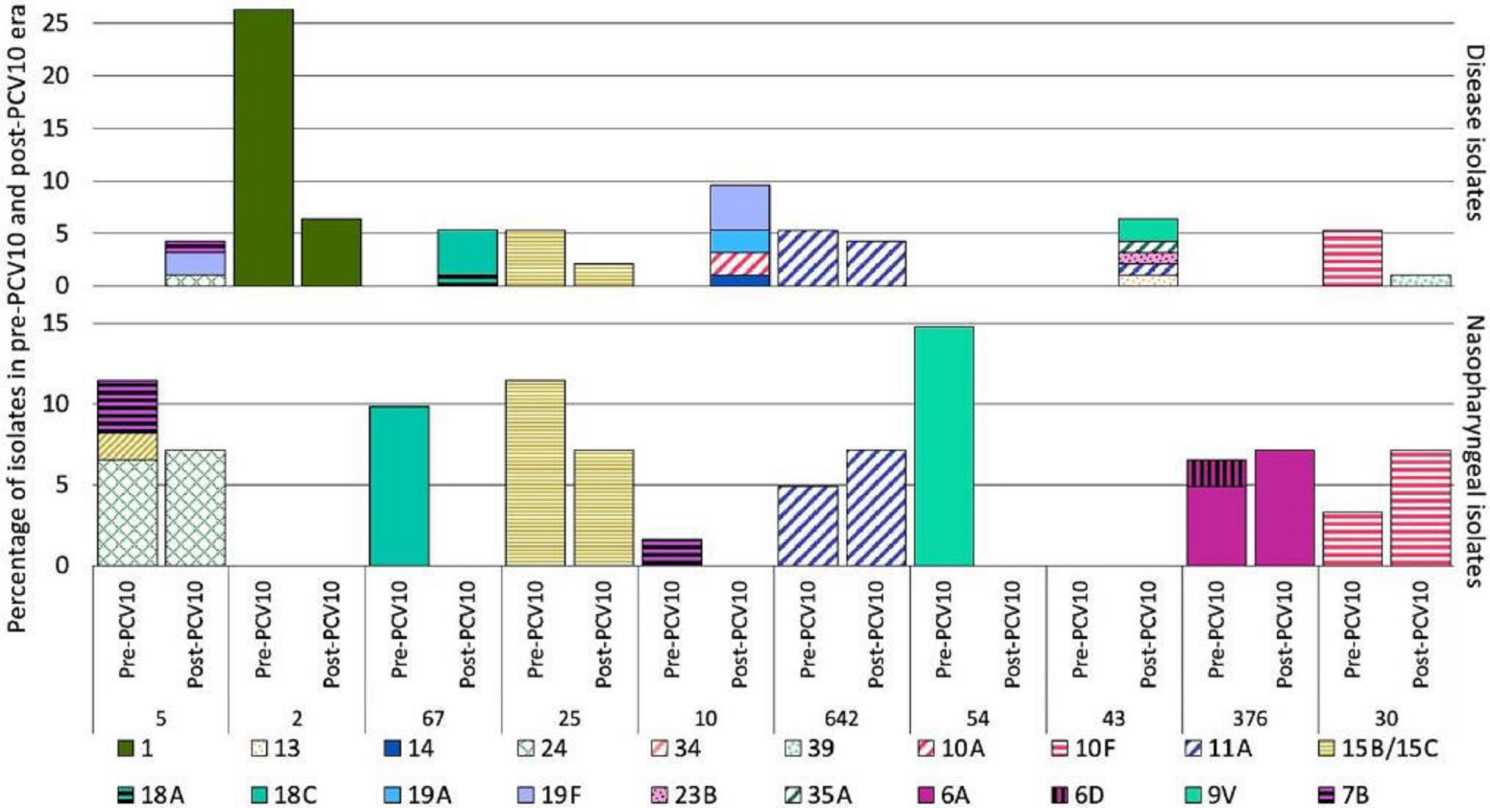
Distribution of serotypes amongst common Global Pneumococcal Sequence Clusters (GPSCs) in the pre- and post-PCV10 era in disease-causing and nasopharyngeal isolates. Prevalent GPSCs with five or more isolates are shown. This figure shows that GPSC5, 10 and 43 are lineages with potential for mediating serotype replacement, because they express a variety of serotypes, are multidrug resistant and are associated with disease-causing samples.

The top five GPSCs accounted for 29 % (55/190) of the isolates in this study. They were GPSC5 (6.38 %; *n*=12), 67 (5.85 %; *n*=11), 25 (5.85 %; *n*=11), 2 (5.85 %; *n*=11) and 10 (5.32 %; *n*=10). The GPSCs varied between disease-causing and nasopharyngeal isolates; the most prevalent GPSCs in the 75 nasopharyngeal isolates were GPSC54 (12%, *n*=9, expressing serotype 9V), 5 (10.67 %; *n*=8; serotypes 15A and 7B, and serogroup 24), 25 (10.67 %; *n*=8, serotype 15B/15C), 67 (8 %; *n*=6; serotype 18C) and 376 (6.67 %; *n*=5; serotypes 6A and 6D). The most common GPSCs in the 113 disease-causing isolates were GPSC2 (9.73%, *n*=11; serotype 1), 10 (7.96 %; *n*=9; serotypes 14, 10A, 19A and 19F), 43 (5.31 %, *n*=6; serotypes 13, 11A, 23B, 35A and 9V), 67 (4.42 %, *n*=5; serotypes 18A and 18C) and 642 (4.42 %, *n*=5; serotype 11A) (Fig. 2 and Table S4). Serotypes found to be associated with disease, including serotypes 1 and 19F, exhibited a difference in their GPSC background. All serotype 1 isolates were expressed by GPSC2, whereas a range of GPSCs expressed serotype 19F (GPSC5, 10, 48, 500 and 958) (Fig S2). On the other hand, serotype 9L which was primarily identified in nasopharyngeal isolates belonged to GPSC650, 112 and 26 (Fig S2). Our results also showed that a significantly higher proportion of GPSC54 (expressing serotype 9V) was observed in nasopharyngeal isolates as compared to disease-causing isolates (Fig. S3). As indicated in Fig. S3, no significant association was observed between GPSCs and the patients’ age and gender. The diversity of GPSCs detected in children and adults was comparable with 43 GPSCs detected in children and 39 in adults. The most prevalent GPSCs in disease isolates in children were GPSC10 (*n*=7) and 5 (*n*=3) whereas GPSC2 (*n*=9), 43 (*n*=5), 67 (*n*=4) and 642 (*n*=4) were the most prevalent in disease isolates from adults. The distribution of serotypes amongst GPSCs over the vaccine period is shown in Fig. S4 and stratified by disease-causing and nasopharyngeal isolates in Fig. 2.

### Detection of capsular switching events in Pakistan

We identified two potential capsular switching events that may have occurred within Pakistan. The first is a potential switch between 19F and 14 within GPSC10 (ST650) in Pakistan. All isolates belonging to ST650, including both serotype 19F (*n*=4, collected in 2017 and 2018) and 14 (*n*=1, collected in 2014), in our dataset were disease isolates. The second capsular switching event in Pakistan is observed between 18C and 18A in isolates belonging to GPSC67 (ST1233). In this case, the 18A isolate, collected in 2017, was a disease isolate whereas 18C isolates were from carriage (*n*=3, isolated in 2013) as well as disease (*n*=4, isolated in 2014, 2015, 2016 and 2017) (Table 3). However, it is worth mentioning that there is limited sampling in countries surrounding Pakistan and therefore an origin of these capsular switching events outside of Pakistan may not have been sampled and cannot be ruled out.

**Table 3.** Capsular switching events in the Pakistan dataset

Event observed	ST	Corresponding GPSC	Serotype (*n*)	Description	Interpretation
Serotype switch between 19F and 14 within GPSC10 (ST650)	650	10	14 (*n*=1); 19F (*n*=4)	Serotype 19F (ST650) isolates from Pakistan cluster together with the singleton serotype 14 (ST650) isolate from Pakistan	A potential serotype switching may have occurred in Pakistan
Serotype switch between 18C and 18A within GPSC67 (ST1233)	1233	67	18A (*n*=1); 18C (*n*=7)	18C (ST1233) isolates from Pakistan cluster together and an 18A (ST1233) isolate from Pakistan also groups with the 18C (ST1233) isolates from Pakistan. This is the only 18A isolate in the GPSC67 phylogeny	A potential serotype switching event between 18C and 18A may have occurred in Pakistan

Links to microreact instances for each GPSC: GPSC67: https://microreact.org/project/kE4nHQL7LcJnTCSL4qjRJ7-gpsc67pk. GPSC10: https://microreact.org/project/6xxcuDAMtAt52Jp5u4dmRr-gpsc10pk. GPS database (last accessed on 1 September 2021) and PubMLST database (last accessed on 1 September 2021).

### Distribution of antimicrobial resistance amongst pneumococcal isolates from Pakistan

Antibiotic susceptibilities to 17 antibiotics were predicted from the antimicrobial resistance (AMR) pipeline and phenotypic susceptibilities to 12 antibiotics were available for comparison. While 100 % concordance was observed for amoxicillin, cefuroxime, cefotaxime, ceftriaxone, linezolid and vancomycin, discordance in genotypic and phenotypic resistance profiles was observed for six antibiotics with highest discordance observed for penicillin (24.5 %, 24/98), represented by 22 different Penicillin Binding Proteins (PBP) profiles. Of these 24 pencillin discordant isolates, 22 (91.7 %) were phenotypically susceptible and predicted to be resistant through the CDC AMR pipeline whereas only two (8.3 %) isolates were phenotypically resistant and predicted to be susceptible through the CDC AMR pipeline (Table S5 and Fig. S5).

**Fig. 3.**
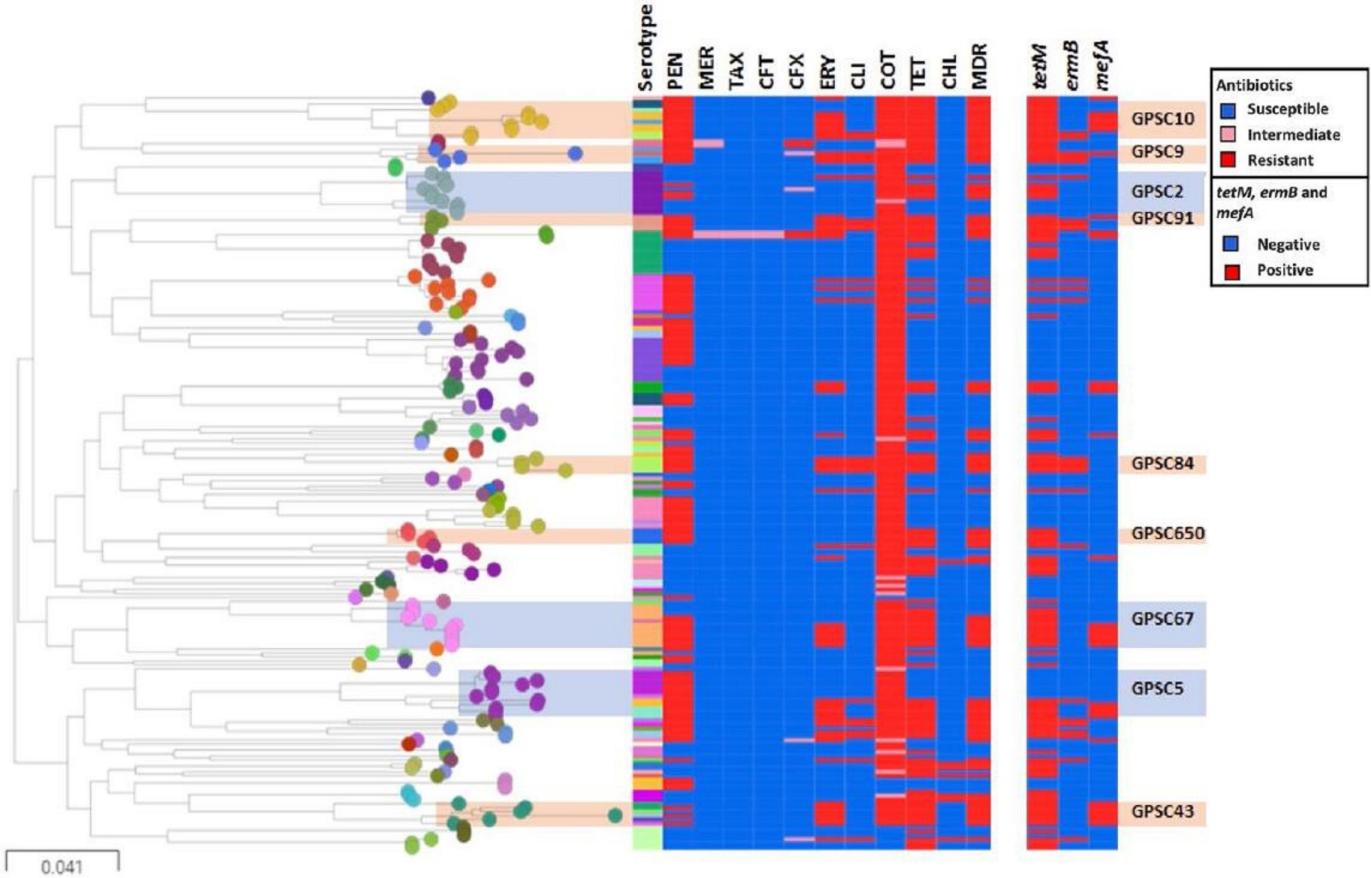
Maximum likelihood tree showing pneumococcal isolates (*n*=190) from Pakistan. A maximum likelihood tree with the isolates from this study (*n*=190) was reconstructed using FastTree. Tree nodes are coloured by GPSCs. *In silico* serotype, resistance profiles and resistance markers are shown in the metadata block. MDR lineages described as GPSCs (with at least four isolates) resistant to three or more groups of antibiotics have been highlighted. GPSCs (GPSC10, 9, 84, 650 and 43) with all MDR isolates are highlighted in orange whereas GPSCs in which only 40–80 % of the isolates are MDR are highlighted in blue (GPSC5, 67 and 2). This tree can be interactively visualized using: https://microreact.org/project/qYqCAfqPSgwyPASKUZ6UFf/d98a9ce5. PEN, penicillin; MER, meropenem; TAX, cefotaxime; CFT, ceftriaxone; CFX, cefuroxime; ERY, erythromycin; CLI, clindamycin; COT, Cotrimoxazole; TET, tetracycline/doxycycline; CHL, chloramphenicol; MDR, multidrug-resistant. Figure colour keys are according to Microreact. 0.041 indicates the difference in nucleotides or the amount of nucleotide change.

**Fig. 4.**
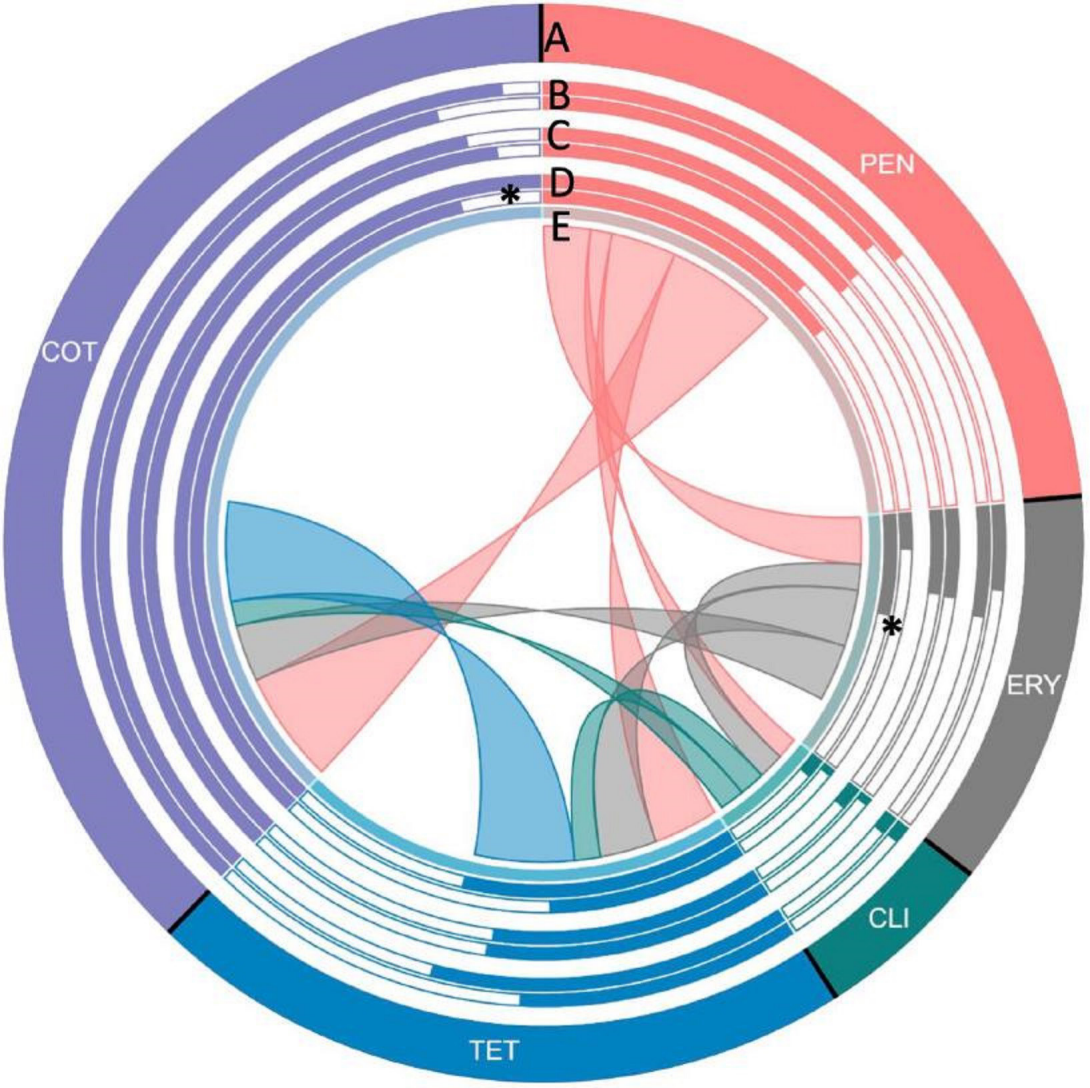
Antimicrobial resistance in pneumococcal isolates in Pakistan. A: overall resistance; B: resistance proportion in children ≤5 years (outer) and those over 5 years (inner); C: resistance proportion in women (outer) and men (inner); D: resistance proportion in nasopharyngeal (outer) and disease-causing isolates (inner); and E: proportion of coresistance. Five antibiotics against which the highest number of isolates were resistant are shown. Each section of the diagram represents the resistance observed in pneumococcal isolates against the antibiotic. Size of each section is proportional to the proportion of resistant isolates to the antibiotic in the study. The bars run in a clockwise direction. B: bar charts showing the proportion of resistant strains in patients of different age groups. The outer and inner bars represent the proportion of resistant isolates from children <5 years of age and individuals over 5 years, respectively. C: gender-wise comparison of susceptibility to resistant pneumococci. Outer and inner circles show the proportion of resistant pneumococcal isolates from women vs. men, respectively. D: comparison among site of isolation. Outer circle and inner circle show the proportion of resistant pneumococci isolated from nasopharyngeal vs. disease-causing isolates, respectively. E: proportion of isolates resistant to one antimicrobial and also resistant to another antimicrobial, as shown by the connections. The are covered by the connection on E is proportional to the level of co-resistance observed. Co-resistance proportions were scaled down to one-fifth of the actual overlap for clear visualization. PEN, penicillin; ERM, erythromycin; CLI, clindamycin; TET, tetracycline/doxycycline; COT, cotrimoxazole.

A phylogenetic tree overlaid with *in silico* inference of AMR, MDR and resistance markers is shown in Fig. 3 and overlaid with vaccine coverage of MDR lineages in Fig. S6. Highest resistance levels were observed against commonly used antibiotics including penicillin (58.9 %, *n*=122), erythromycin (29.5 %, *n*=56), clindamycin (13.2 %, *n*=25), co-trimoxazole (94.2 %, *n*=179) and tetracycline/doxycycline (53.2 %, *n*=101) (Fig. 4 and Table 4). Low levels of resistance were observed against extended-spectrum β-lactams (1.1 –4.2 %) whereas all isolates were susceptible to vancomycin, rifampicin, levofloxacin, linezolid, quinupristin/dalfopristin and amoxicillin. A higher proportion of disease-causing isolates were MDR as compared to nasopharyngeal isolates (54 % vs 25.3 %; *P*=0.0011) as shown in Table 4. First-line antibiotic penicillin-resistant lineages which were MDR included over 25 GPSCs (*n*=69). The most prevalent penicillin-resistant MDR GPSCs included GPSC10 (*n*=9), 67 (*n*=7), 43 (*n*=5), 5 (*n*=5), 84 (*n*=4) and 91 (*n*=4). Further, we observed a lower percentage of resistance against erythromycin in nasopharyngeal isolates as compared to disease-causing isolates (13.3 % vs. 39.9 %; *P*=0.0014) whereas resistance to co-trimoxazole was higher in nasopharyngeal isolates (100 % vs. 90.3 %; *P*=0.0246) as compared to disease-causing isolates (Table 4 and Fig. 3). No significant association between patient demographics and resistance could be established (Table S6 and Fig. 4).

**Table 4.** Antimicrobial resistance of the pneumococcal collection from nasopharynx swabs (*n*=75), pneumococcal diseases (*n*=113) and total isolates (*n*=190), Pakistan, 2006–2020

Antibiotic	Site of isolation			Total
	Nasopharyngeal	Disease-causing	*P* for difference	
Co-trimoxazole	100 % (75/75)	90.3 % (102/113)	0.0194	94.2 % (179)
Penicillin	53.3 % (40/75)	61.9 % (70/113)	0.6386	58.9 % (112)
Tetracycline	41.3 % (31/75)	60.2 % (68/113)	0.0614	53.2 % (101)
Doxycycline	41.3 % (31/75)	60.2 % (68/113)	0.0614	53.2 % (101)
Erythromycin	13.3 % (10/75)	38.9 % (44/113)	0.00109	29.5 % (56)
Clindamycin	5.3 % (4/75)	16.8 % (19/113)	0.07001	15.3 % (25)
Cefuroxime	1.3 % (1/75)	6.2 % (7/113)	0.3712	4.2 % (8)
Chloramphenicol	4 % (3/75)	3.5 % (4/113)	1	3.7 % (7)
Meropenem	0 % (0/75)	3.5 % (4/113)	0.3712	2.1 % (4)
Cefotaxime	0 % (0/75)	1.8 % (2/113)	0.95	1.1 % (2)
Ceftriaxone	0 % (0/75)	1.8 % (2/113)	0.95	1.1 % (2)
Multidrug resistance	25.3 % (19/75)	54 % (61/113)	0.0011	43.1 % (82)

No statistical difference was detected in age and gender wise comparisons.

In the context of GPSCs, all isolates belonging to GPSC10, 9, 91, 84, 650 and 43 were MDR. On the other hand, 40–80 % of the isolates belonging to GPSC2 (45.4 %, 5/11), 5 (41.7 %, 5/12) and 67 (72.7 %, 8/11) were MDR. This MDR was mediated mainly through resistance against co-trimoxazole, tetracycline, penicillin and erythromycin in these three GPSCs. All GPSC2 MDR isolates were resistant to tetracycline and co-trimoxazole, four isolates were resistant to beta-lactams and one isolate was resistant to macrolides. On the other hand, all MDR isolates in GPSC5 and 67 were resistant to penicillin, erythromycin, cotrimoxazole and tetracycline (Fig. 3).

## Discussion

Recognizing that PCV10 has been part of the EPI in Pakistan since October 2012 [3], we report here for the first time on the biology of the pneumococcal population from Pakistan between 2006 and 2020 using whole genome sequencing data.

Our serotyping results suggest that PCV10 covers a low proportion of circulating pneumococci in Pakistan (proportion of isolates expressing PCV10 serotypes). The proportion of isolates of serotypes included in PCV10 in disease-causing isolates was lower in the post-PCV10 era (33.3 %; *n*=31/93) as compared to the pre-PCV10 era (47.4 %; *n*=9/19). As observed in previous studies, we found that 19F and serotype 1 continued to cause infections years after the introduction of PCV10. Interestingly, a similar proportion (47.1 %) of isolates responsible for causing acute lower respiratory infections in children under 5 years in Pakistan were of serotypes included in PCV10 [5]. In more recent surveys, carriage and disease-causing isolates from children demonstrate 30.7–61% coverage by PCV10 [7–10]. Similar to our results, these studies also report only a slightly higher coverage rate by PCV13 [7–10]. The most prevalent NVTs (serotypes not covered by PCV10) in the post-vaccine era, overall and in the disease-causing isolates, in our study included 6A, 3, 19A, 23B, 10A and 11A. Of these six serotypes, 6A, 3 and 19A are included in PCV13, which was recently introduced in the EPI in Pakistan. The introduction of these six most prevalent NVTs in PCV10 would increase overall coverage rates by 30.9 %. Of the current vaccines in development, PCV24 would provide highest coverage of 72.4 % in disease-causing isolates from children and 70.2 % in disease-causing isolates in all age groups in the post-vaccine era.

We report high resistance levels against erythromycin, co-trimoxazole, penicillin and tetracycline in the post-PCV10 era among disease-causing isolates, which can also be explained on the basis of increased antimicrobial usage. In recent years, Pakistan has seen an increased usage of macrolides with total erythromycin usage reaching 247.5 defined daily dosage (DDD) in December 2017, β-lactams (total DDD of 412.8 for amoxicillin-clavulanic acid) and tetracycline (total DDD of 24 in 2016) [27–31] which has been associated with increased resistance rates in major pathogens [32]. Another study which investigated AMR in pneumococcal isolates from children under the age of 2 years also reported high levels of resistance against macrolides, co-trimoxazole and tetracycline in the post-PCV10 era [33]. While vaccination may have helped in lowering infection rates caused by vaccine serotypes, this excessive usage of antimicrobials may have circumvented the beneficial impact of vaccine introduction on lowering resistance rates.

Major globally spreading high-risk GPSCs associated with disease include GPSC10, 2, 43 and 5, and these were noted in our dataset, as well [11]. However, surprisingly, GPSC1, which is one of the high-risk globally spreading GPSCs and also reported as the most prevalent GPSC in neighbouring India [11, 23, 34], was not detected among the isolates we evaluated. While the number of isolates that we tested was relatively small, these findings highlight that the pneumococcal population in Pakistan may be distinct from other regions as well as neighbouring countries in South Asia.

Our study is the first to characterize the pneumococcal population using whole genome sequencing data from Pakistan; however, the study has its limitations. First, the study sample size was relatively small; thus, we did not have the power to make conclusions pertaining to pre- and post-vaccine era differences in the distribution of serotypes and genotypes. In addition, as the information on clinical manifestations and the patients’ immune status was not available for disease-causing isolates, we could not determine an association of a particular serotype/genotype with clinical manifestation. Future studies with information on disease manifestation and the patients’ immune status coupled with a greater number of isolates would be helpful in better understanding pneumococcal strains from Pakistan.

In conclusion, this study reports that PCV10 has limited potential coverage against pneumococcal infection in Pakistan. The high diversity of serotypes argues for a vaccine with a substantially higher number of serotypes (e.g. PCV24/25). We also report a high level of diversity at the genotypic level. In comparison with the pre-PCV10 era isolates, we observed a higher proportion of MDR isolates in the post-PCV10 era in disease-causing isolates. Further study with a collection of larger sample size and an extended temporal window is needed for an in-depth understanding of the pneumococcal population in Pakistan in the post-vaccine era.

## Supplementary Data

Supplementary material 1

Supplementary material 2

## References

[1] Henriques-Normark B, Tuomanen EI (2013). The pneumococcus: epidemiology, microbiology, and pathogenesis. Cold Spring Harb Perspect Med.

[2] Wahl B, O’Brien KL, Greenbaum A, Majumder A, Liu L (2018). Burden of *Streptococcus pneumoniae* and *Haemophilus* influenzae type b disease in children in the era of conjugate vaccines: global, regional, and national estimates for 2000-15. Lancet Glob Health.

[3] Riaz A, Mohiuddin S, Husain S, Yousafzai MT, Sajid M (2019). Effectiveness of 10-valent pneumococcal conjugate vaccine against vaccine-type invasive pneumococcal disease in Pakistan. Int J Infect Dis.

[4] (2022). Pneumococcal vaccination coverage. https://immunizationdata.who.int/pages/coverage/PCV.html?CODE=PAK&ANTIGEN=PCV3&YEAR=.

[5] Mastro TD, Spika JS, Facklam RR, Thornsberry C, Ghafoor A (1991). Antimicrobial resistance of pneumococci in children with acute lower respiratory tract infection in Pakistan. The Lancet.

[6] Nisar MI, Ahmed S, Jehan F, Shahid S, Shakoor S (2021). Direct and indirect effect of 10 valent pneumococcal vaccine on nasopharyngeal carriage in children under 2 years of age in Matiari, Pakistan. Vaccine.

[7] Nisar MI, Nayani K, Akhund T, Riaz A, Irfan O (2019). Nasopharyngeal carriage of Streptococcus pneumoniae in children under 5 years of age before introduction of pneumococcal vaccine (PCV10) in urban and rural districts in Pakistan. BMC Infect Dis.

[8] Khan F, Khan MA, Ahmed N, Khan MI, Bashir H (2018). Molecular characterization of pneumococcal surface protein A (PspA), Serotype Distribution and Antibiotic susceptibility of Streptococcus pneumoniae strains isolated from Pakistan. Infect Dis Ther.

[9] Shakoor S, Kabir F, Khowaja AR, Qureshi SM, Jehan F (2014). Pneumococcal serotypes and serogroups causing invasive disease in Pakistan, 2005-2013. PLoS One.

[10] Bibi S, Siddiqui TR, Hassan SF, Ahmed W (2017). Report - Colonization rates and immune status of children against Streptococcus pneumoniae before the introduction of pneumococcal conjugate vaccine. Pak J Pharm Sci.

[11] Gladstone RA, Lo SW, Lees JA, Croucher NJ, van Tonder AJ (2019). International genomic definition of pneumococcal lineages, to contextualise disease, antibiotic resistance and vaccine impact. EBioMedicine.

[12] Lo SW, Gladstone RA, van Tonder AJ, Lees JA, du Plessis M (2019). Pneumococcal lineages associated with serotype replacement and antibiotic resistance in childhood invasive pneumococcal disease in the post-PCV13 era: an international whole-genome sequencing study. Lancet Infect Dis.

[13] (2022). Final Results (Census-2017) | Pakistan Bureau of Statistics. https://www.pbs.gov.pk/content/final-results-census-2017.

[14] CLSI (2018). M100 Performance Standards for Antimicrobial Susceptibility Testing A CLSI supplement for global application. www.clsi.org.

[15] Epping L, van Tonder AJ, Gladstone RA, Bentley SD (2018). SeroBA: rapid high-throughput serotyping of Streptococcus pneumoniae from whole genome sequence data. Microb Genom.

[16] J. Page A, Taylor B, A. Keane J (2016). Multilocus sequence typing by blast from de novo assemblies against PubMLST. JOSS.

[17] Li Y, Metcalf BJ, Chochua S, Li Z, Gertz RE (2016). Penicillin-binding protein transpeptidase signatures for tracking and predicting β-lactam resistance levels in Streptococcus pneumoniae. mBio.

[18] Metcalf BJ, Chochua S, Gertz RE, Li Z, Walker H (2016). Using whole genome sequencing to identify resistance determinants and predict antimicrobial resistance phenotypes for year 2015 invasive pneumococcal disease isolates recovered in the United States. Clin Microbiol Infect.

[19] Li Y, Metcalf BJ, Chochua S, Li Z, Gertz RE (2017). Validation of β-lactam minimum inhibitory concentration predictions for pneumococcal isolates with newly encountered penicillin binding protein (PBP) sequences. BMC Genomics.

[20] Lees JA, Harris SR, Tonkin-Hill G, Gladstone RA, Lo SW (2019). Fast and flexible bacterial genomic epidemiology with PopPUNK. Genome Res.

[21] Price MN, Dehal PS, Arkin AP (2009). FastTree: computing large minimum evolution trees with profiles instead of a distance matrix. Mol Biol Evol.

[22] Argimón S, Abudahab K, Goater RJE, Fedosejev A, Bhai J (2016). Microreact: visualizing and sharing data for genomic epidemiology and phylogeography. Microb Genom.

[23] Nagaraj G, Govindan V, Ganaie F, Venkatesha VT, Hawkins PA (2021). *Streptococcus pneumoniae* genomic datasets from an Indian population describing pre-vaccine evolutionary epidemiology using a whole genome sequencing approach. Microb Genom.

[24] Li H, Durbin R (2009). Fast and accurate short read alignment with burrows-Wheeler transform. Bioinformatics.

[25] Croucher NJ, Page AJ, Connor TR, Delaney AJ, Keane JA (2015). Rapid phylogenetic analysis of large samples of recombinant bacterial whole genome sequences using Gubbins. Nucleic Acids Res.

[26] van Selm S, van Cann LM, Kolkman MAB, van der Zeijst BAM, van Putten JPM (2003). Genetic basis for the structural difference between Streptococcus pneumoniae serotype 15B and 15C capsular polysaccharides. Infect Immun.

[27] Khan EA Situation Analysis Report on Antimicrobial Resistance in Pakistan-Findings and Recommendations for Antibiotic Use and Resistance. Internet] The Global Antibiotic Resistance Partnership (GARP), Pakistan. https://wwwcddep%20org/publications/garp-pakistan-situation-analysis.

[28] Hayat K, Rosenthal M, Gillani AH, Chang J, Ji W (2019). Perspective of key healthcare professionals on antimicrobial resistance and stewardship programs: a multicenter cross-Sectional study from Pakistan. Front Pharmacol.

[29] Ali H, Zafar F, Alam S, Beg AE, Bushra R (2018). Drug utilization and prescribing pattern of antibiotics in a tertiary care setups; trends and practices. Pak J Pharm Sci.

[30] Saleem Z, Faller EM, Godman B, Malik MSA, Iftikhar A (2021). Antibiotic consumption at community pharmacies: a multicenter repeated prevalence surveillance using WHO methodology. Med Access Point Care.

[31] National AMR Action Plan for Pakistan Antimicrobial Resistance National Action Plan Pakistan Ministry of National Health Services Regulations & Coordination Government of Pakistan.

[32] Javaid N, Sultana Q, Rasool K, Gandra S, Ahmad F (2021). Trends in antimicrobial resistance amongst pathogens isolated from blood and cerebrospinal fluid cultures in Pakistan (2011-2015): a retrospective cross-sectional study. PLoS One.

[33] Nisar MI, Shahid S, Jehan F, Ahmed S, Shakoor S (2021). Antimicrobial resistance in pneumococcal carriage isolates from children under 2 years of age in Rural Pakistan. Microbiol Spectr.

[34] Corcoran M, Mereckiene J, Cotter S, Murchan S, Lo SW (2021). Using genomics to examine the persistence of Streptococcus pneumoniae serotype 19A in Ireland and the emergence of a sub-clade associated with vaccine failures. Vaccine.

