# Supplementary material for: Strain features of pneumococcal isolates in the pre- and post-PCV10 era in Pakistan
Source: Microb Genom. 2024 Jan 25;10(1):001163. doi: 10.1099/mgen.0.001163 (PMC10868622; doi:10.1099/mgen.0.001163)
Supplement: Supplementary material 1 [file mgen-10-1163-s001.pdf]

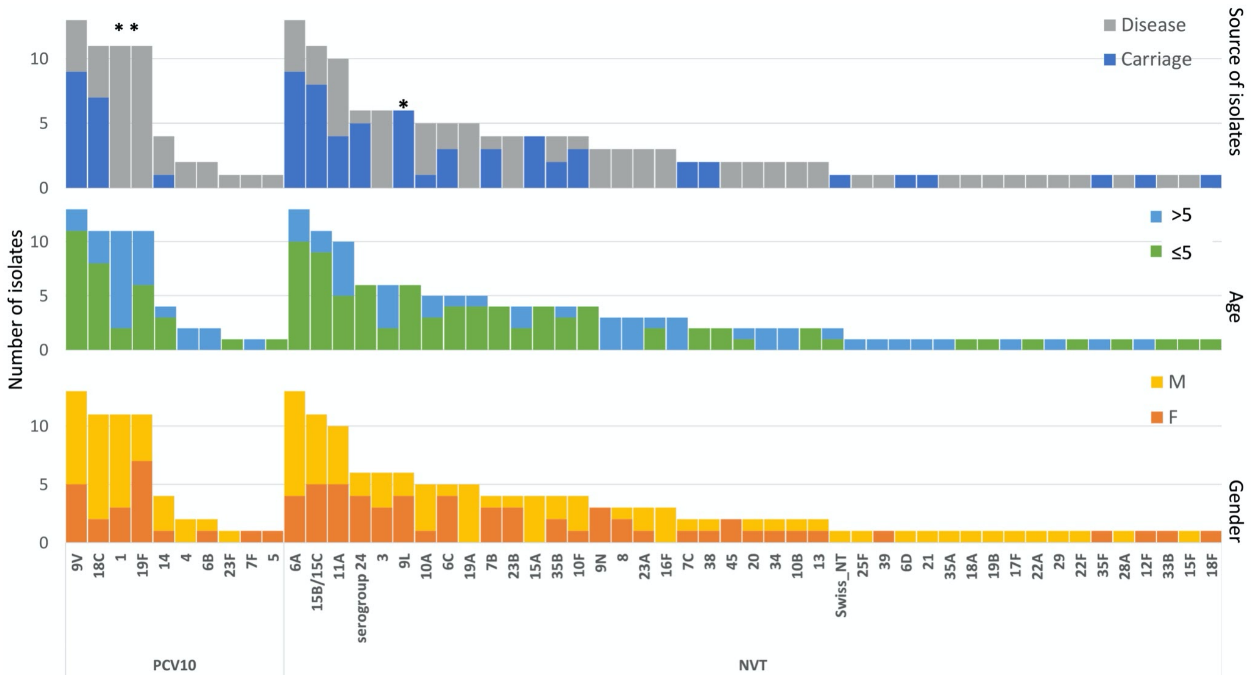

Fig. S1. **Distribution of serotypes over source of isolates, patients' age, and patients' gender in pneumococcal isolates from Pakistan.** All serotypes (n=53) from all isolates included in this study are shown. This figure shows that higher proportions of serotype 1 and 19F were isolated from disease-causing samples as compared to nasopharyngeal samples whereas a higher proportion of 9L was isolated from nasopharyngeal samples. No significant difference was detected in age and gender-wise comparisons.

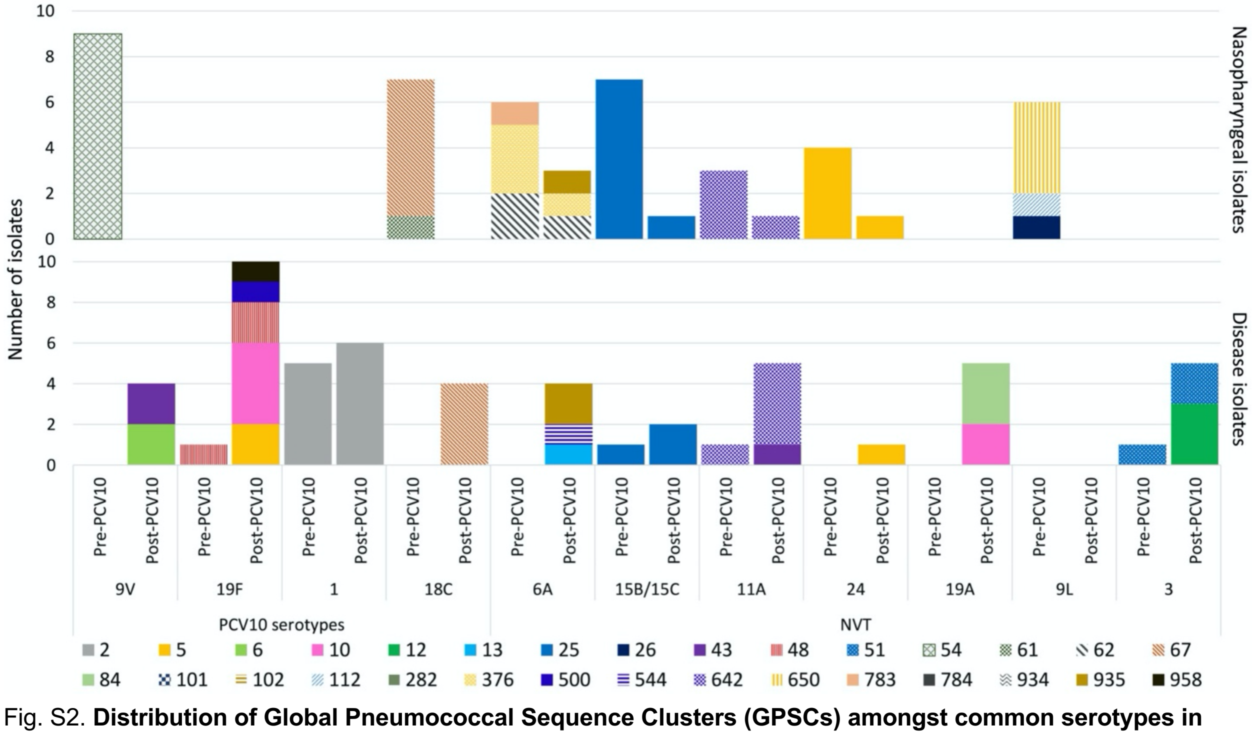

Fig. S2. **Distribution of Global Pneumococcal Sequence Clusters (GPSCs) amongst common serotypes in pre- and post-PCV10 era in disease-causing and nasopharyngeal isolates.** Prevalent serotypes with six or more isolates are shown here. This figure shows that serotype PCV10 19F and NVT 6A (the most prevalent NVT serotype) are one of the important serotypes as they are expressed by a variety of GPSCs.

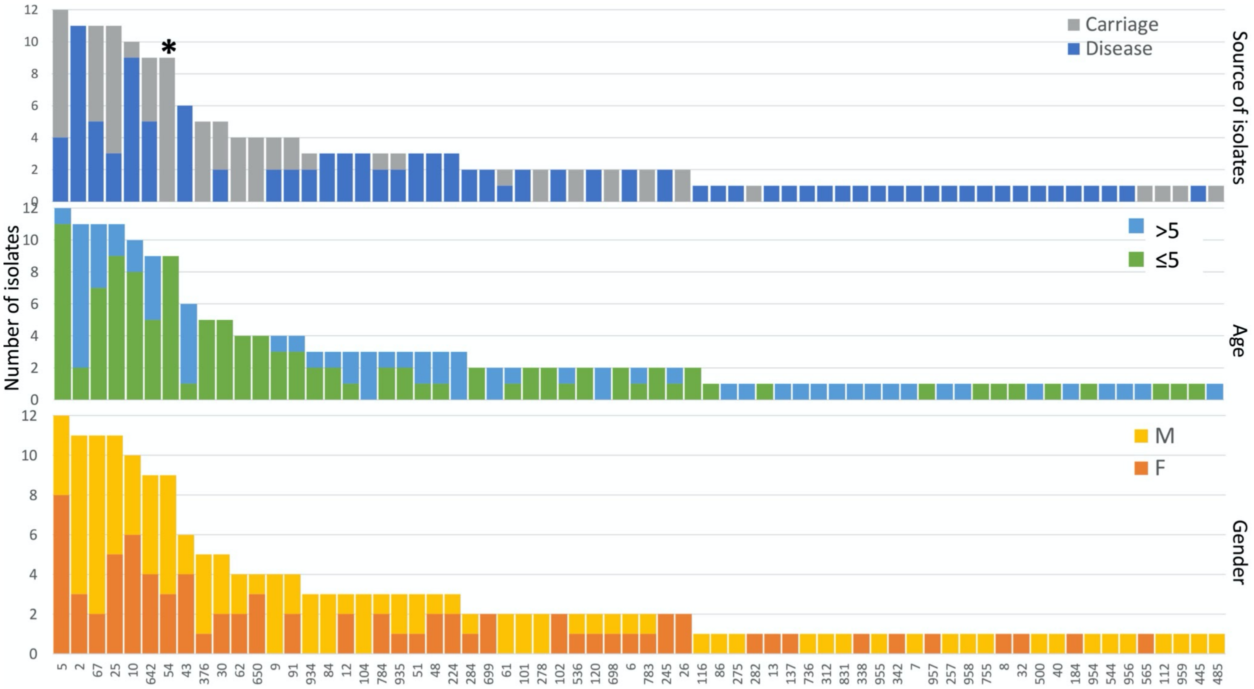

Fig. S3. **Distribution of Global Pneumococcal Sequence Clusters (GPSCs) over source of isolates, age, and gender in isolates from Pakistan.** All GPSCs (n=66) from all nasopharyngeal as well as disease-causing isolates in this study are shown. This figure shows that a higher proportion of GPSC54 was isolated from nasopharyngeal samples as compared to the disease-causing samples.

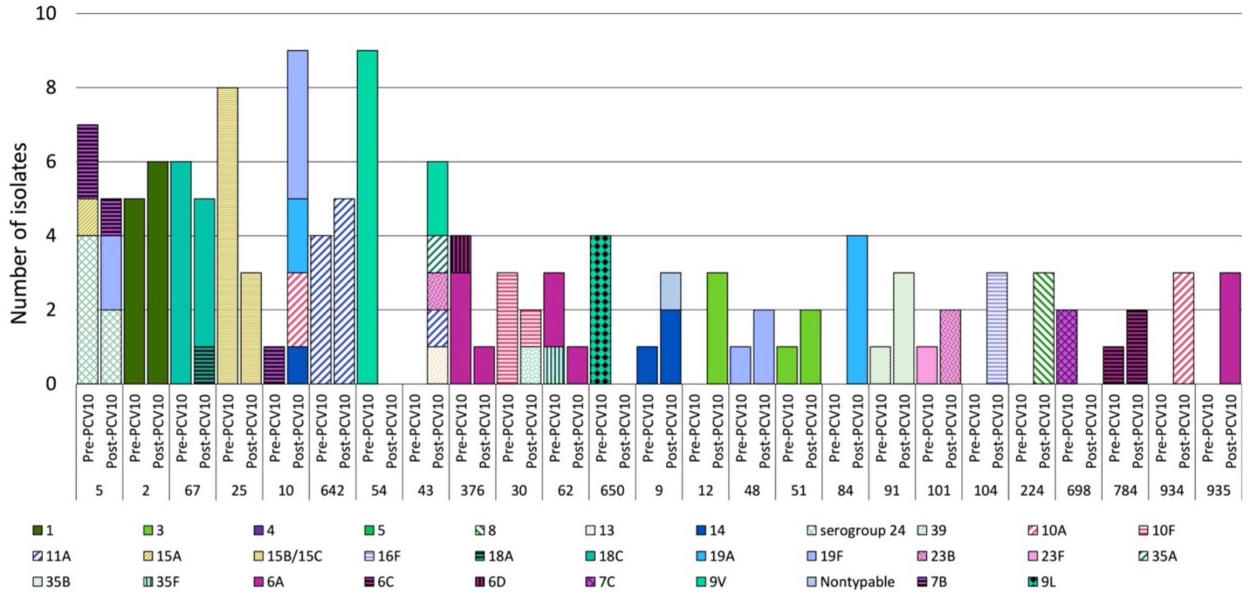

Fig. S4. **Distribution of serotypes amongst common Global Pneumococcal Sequence Clusters (GPSCs) in pre- and post-PCV10 era in isolates from Pakistan.** Prevalent GPSCs with three or more isolates are shown from all pneumococcal isolates (including nasopharyngeal and disease-causing isolates) in this study. This figure shows that GPSC5, 10, and 43 are the most important lineages because they are multidrug resistant and express both vaccine and non-vaccine serotypes.

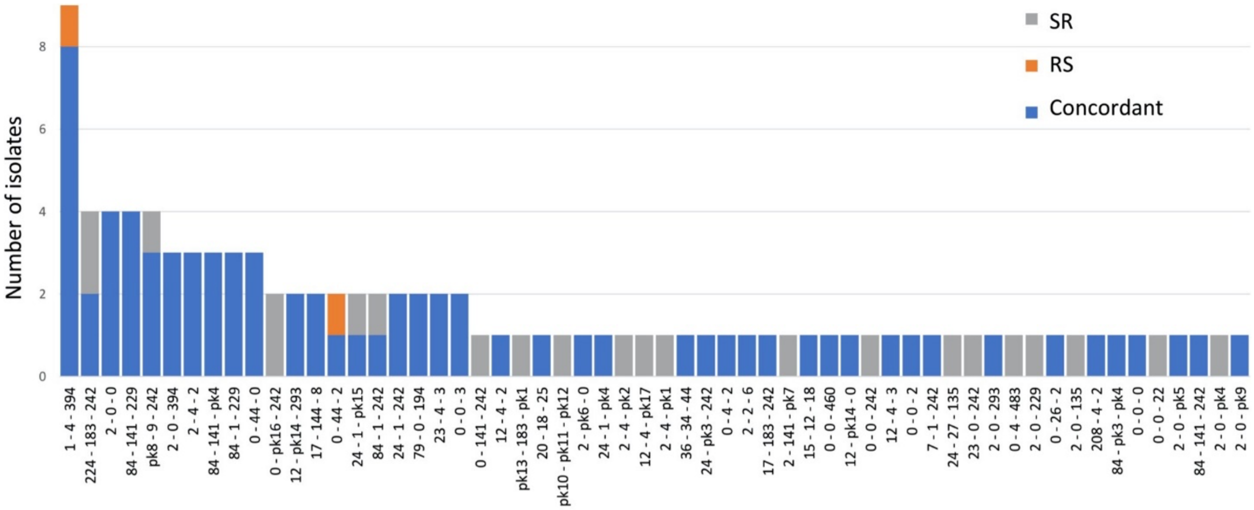

Fig. S5. **Distribution of pbp profiles of pneumococcal isolates overlaid with concordance between in silico and phenotypic penicillin resistance**. SR: Phenotypically susceptible and predicted resistant through CDC AMR pipeline; RS: Phenotypically resistant and predicted susceptible through CDC AMR pipeline; and concordant: where the phenotypic and genotypic results were concordant. Pbp numbers with “pk '' were not detected in the PBP database (https://www.cdc.gov/streplab/pneumococcus/mic.html) and were numbered sequentially on the basis of sequence differences.

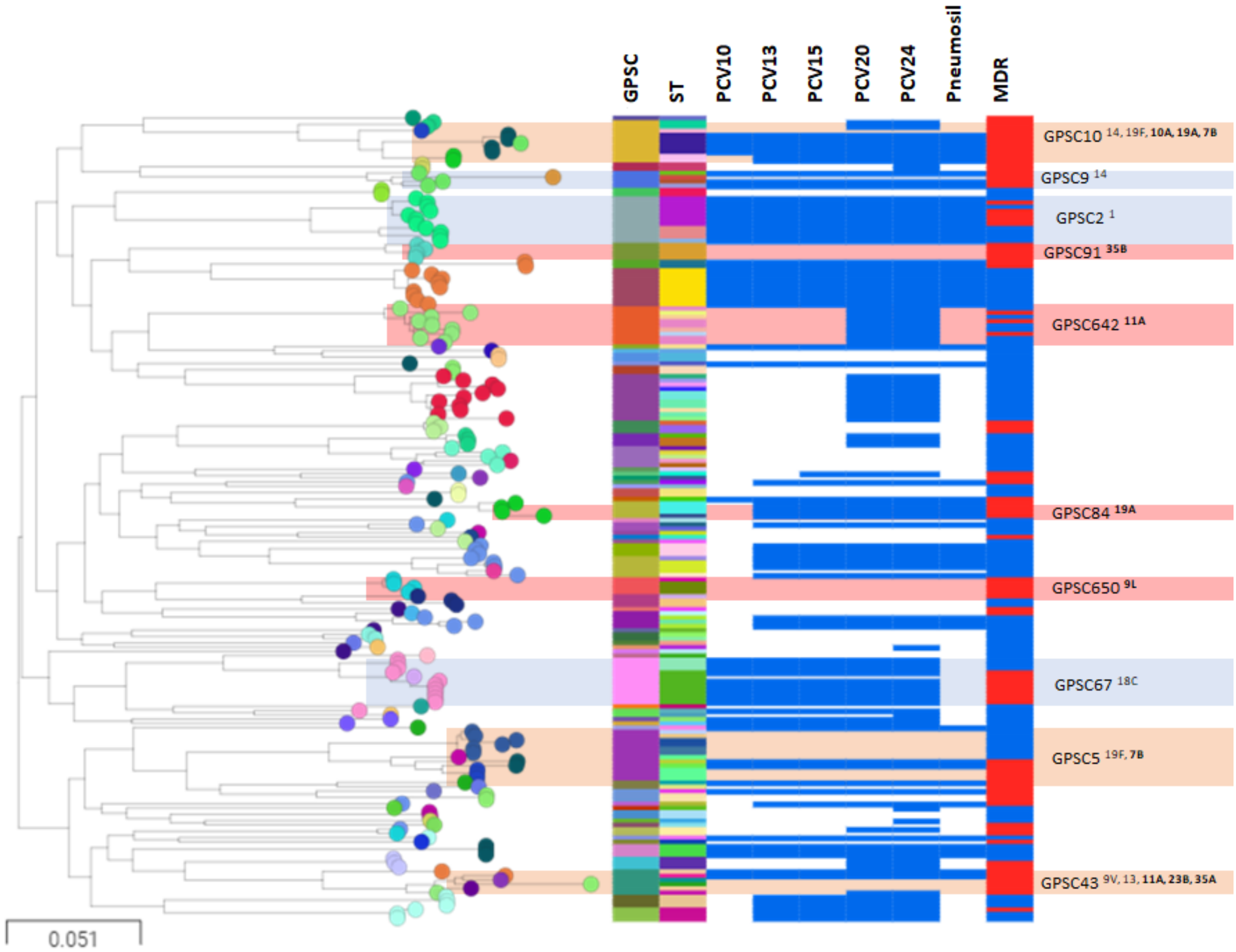

Fig. S6. **Maximum likelihood tree showing vaccine coverage of multidrug resistant (MDR) pneumococcal isolates from Pakistan by different vaccines**. A maximum likelihood tree with the isolates from this study (n=190) was constructed using FastTree. Tree nodes are coloured by *in silico serotypes*. GPSCs, sequence types (ST), multidrug resistance, and coverage of pneumococcal conjugate vaccines PCV10, PCV13, PCV15, PCV20, PCV24, and pneumosil are shown in metadata block. For vaccine coverage, blue colour indicates that the serotype expressed by the isolate is covered by the given vaccine whereas transparent metadata block entry indicates that the serotype is not covered by the given vaccine. For MDR, Red colour indicates that the isolates are MDR and blue colour indicates that the isolates non-MDR. Multidrug resistant lineages have been highlighted. Blue highlight blocks show that all serotypes expressed by the MDR-GPSC is covered by PCV10, red indicates that none of the serotypes expressed by the GPSC is covered by PCV10, and orange colour indicates that the GPSC expresses both PCV10 and non-PCV10 serotypes. This tree can be interactively visualized using: https://microreact.org/project/qYqCAfqPSgwyPASKUZ6UFf/77268fff. While PCV10, PCV13, and pneumosil are currently available in the market, PCV15 is under review for the use of individuals over 18 years old, PCV20 is approved by FDA to be used for individuals over 18 years old and PCV24 is still under development. GPSC, global pneumococcal sequencing cluster; ST, sequence type; MDR, multidrug-resistant. . Figure colour legends are presented in the microreact instance.

Table S2. Distribution of pneumococcal disease-causing isolates over sites of isolation.

Site of isolation	Number of isolates (%)
Blood	35 (31%)
Sputum	22 (19.5%)
Pus	17 (15%)
Cerebrospinal fluid	13 (11.5%)
Ear Swab	9 (8%)
Pleural fluid	4 (3.5%)
Eye swab	3 (2.7%)
Wound Swab	2 (1.8%)
Tracheal Secretion	2 (1.8%)
Bronchial lavage	2 (1.8%)
Ascitic Fluid	2 (1.8%)
Neck abscess	1 (0.9%)
Fluid [a]	1 (0.9%)

[a] For one isolates, only fluid was given in the site of isolation. It was added as it is to the table.

Table S3. Distribution of serotypes in pneumococcal isolates over disease-causing, nasopharyngeal isolates, and the complete dataset, respectively.

Vaccine type	Serotype	Nasopharyngeal isolate (n = 75)	Disease-causing Isolates (n = 113)	All isolate (n=190)
PCV10	5	-	0.8% (1)	0.5% (1)
	1	-	9.7% (11)	5.8% (11)
	7F	-	0.8% (1)	0.5% (1)
	14	1.3% (1)	2.7% (3)	3.5% (4)

	19F	-	9.7% (11)	5.8% (11)
	6B	-	1.8% (2)	1.1% (2)
	23F	-	0.8% (1)	0.5% (1)
	9V	12.0% (9)	3.5% (4)	6.8% (13)
	18C	9.3% (7)	3.5% (4)	5.8% (11)
	4	-	1.8% (2)	1.1% (2)
PCV13-additional	6A	12.0% (9)	3.5% (4)	6.8% (13)
	3	-	5.5% (6)	3.2% (6)
	19A	-	4.4% (5)	3.2% (6)
NVT	15B/15C	10.7% (8)	2.7% (3)	5.8% (11)
	15A	5.3% (4)	-	2.1% (4)
	7C	2.7% (2)	-	1.1% (2)
	10B	-	1.8% (2)	1.1% (2)
	serogroup 24	6.7% (5)	0.8% (1)	3.2% (6)
	9L	8.0% (6)	-	8.0% (5)
	7B	4.0% (3)	0.8% (1)	2.1% (4)
	38	2.7% (2)	-	1.1% (2)
	16F	-	2.7% (3)	1.6% (3)
	6C	4.0% (3)	1.8% (2)	2.6% (5)
	35B	2.7% (2)	1.8% (2)	2.1% (4)
	23A	-	2.7% (3)	1.6% (3)
	10F	4.0% (3)	0.8% (1)	2.1% (4)

	21	1.3% (1)	-	0.5% (1)
	39	-	0.8% (1)	0.5% (1)
	18A	-	0.8% (1)	0.5% (1)
	23B	-	3.5% (4)	2.6% (5)
	28A	-	0.8% (1)	0.5% (1)
	25F	-	0.8% (1)	0.5% (1)
	22A	-	0.8% (1)	0.5% (1)
	35A	-	0.8% (1)	0.5% (1)
	34	-	1.8% (2)	1.1% (2)
	20	-	1.8% (2)	1.1% (2)
	Nontypable	1.3% (1)	-	0.5% (1)
	13	-	1.8% (2)	1.1% (2)
	33B	-	0.8% (1)	0.5% (1)
	29	-	0.8% (1)	0.5% (1)
	19B	-	0.8% (1)	0.5% (1)
	9N	-	2.7% (3)	1.6% (3)
	15F	-	0.8% (1)	0.5% (1)
	17F	-	0.8% (1)	0.5% (1)
	45	-	1.8% (2)	1.1% (2)
	6D	1.3% (1)	-	0.5% (1)
	18F	1.3% (1)	-	0.5% (1)
	35F	1.3% (1)	-	0.5% (1)
	22F	-	0.8% (1)	0.5% (1)
	10A	1.3% (1)	3.5% (4)	2.6% (5)
	11A	5.3% (4)	5.5% (6)	5.3% (10)

	8	-	2.7% (3)	1.6% (3)
	12F	1.3% (1)	-	0.5% (1)

There were two isolates for which we did not have information on the site of isolation. These isolates are included in the all isolates column but not included in the disease and carriage columns.

Table S4. Changes in GPSC distribution in Pakistan over nasopharyngeal, disease-causing, and all isolates, respectively.

GPSC	Nasopharyngeal isolates (n = 75)	Disease-causing isolates (n=113)	All isolates (n=190)
54	12.0% (9)	-	4.7% (9)
25	10.7% (8)	2.7% (3)	5.8% (11)
5	10.7% (8)	3.5% (4)	6.3% (12)
67	8.0% (6)	4.4% (5)	5.8% (11)
2	-	9.7% (11)	5.8% (11)
642	5.3% (4)	4.4% (5)	4.7% (9)
376	6.7% (5)	-	2.6% (5)
650	5.3% (4)	-	2.1% (4)
30	4.0% (3)	1.8% (2)	2.6% (5)
62	5.3% (4)	-	2.1% (4)
26	2.7% (2)	-	1.1% (2)
245	-	1.8% (2)	1.1% (2)
278	2.7% (2)	-	1.1% (2)

284	-	1.8% (2)	1.1% (2)
536	2.7% (2)	-	1.1% (2)
698	2.7% (2)	-	1.1% (2)
10	1.3% (1)	8.0% (9)	5.3% (10)
9	2.7% (2)	1.8% (2)	2.1% (4)
91	2.7% (2)	1.8% (2)	2.1% (4)
48	-	2.7% (3)	1.6% (3)
51	-	2.7% (3)	1.6% (3)
101	-	1.8% (2)	1.6% (3)
784	1.3% (1)	1.8% (2)	1.6% (3)
61	1.3% (1)	0.8% (1)	1.1% (2)
783	2.7% (2)	-	1.1% (2)
8	-	0.8% (1)	0.5% (1)
112	1.3% (1)	-	0.5% (1)
116	-	0.8% (1)	0.5% (1)
282	1.3% (1)	-	0.5% (1)
485	1.3% (1)	-	0.5% (1)
565	1.3% (1)	-	0.5% (1)

43	-	5.5% (6)	3.2% (6)
84	-	2.7% (3)	2.1% (4)
12	-	2.7% (3)	1.6% (3)
104	-	2.7% (3)	1.6% (3)
224	-	2.7% (3)	1.6% (3)
935	1.3% (1)	1.8% (2)	1.6% (3)
934	1.3% (1)	1.8% (2)	1.6% (3)
6	-	1.8% (2)	1.1% (2)
102	-	1.8% (2)	1.1% (2)
120	-	1.8% (2)	1.1% (2)
699	-	1.8% (2)	1.1% (2)
7	-	0.8% (1)	0.5% (1)
13	-	0.8% (1)	0.5% (1)
32	-	0.8% (1)	0.5% (1)
40	-	0.8% (1)	0.5% (1)
86	-	0.8% (1)	0.5% (1)
137	-	0.8% (1)	0.5% (1)
184	-	0.8% (1)	0.5% (1)

257	-	0.8% (1)	0.5% (1)
275	-	0.8% (1)	0.5% (1)
312	-	0.8% (1)	0.5% (1)
338	-	0.8% (1)	0.5% (1)
342	-	0.8% (1)	0.5% (1)
445	-	0.8% (1)	0.5% (1)
500	-	0.8% (1)	0.5% (1)
544	-	0.8% (1)	0.5% (1)
736	-	0.8% (1)	0.5% (1)
755	-	0.8% (1)	0.5% (1)
831	-	0.8% (1)	0.5% (1)
959	1.3% (1)	-	0.5% (1)
957	-	0.8% (1)	0.5% (1)
956	-	0.8% (1)	0.5% (1)
955	-	0.8% (1)	0.5% (1)
954	-	0.8% (1)	0.5% (1)
958	-	0.8% (1)	0.50.5% (1)

There were two isolates for which we did not have information on the site of isolation. These isolates are included in the all isolates column but not included in the disease and carriage columns.

Table S5. Summary of discordance between predicted and phenotypic antimicrobial susceptibility results

Antibiotics	Discordant results	Insilico R and phenotypic S	Insilico S and phenotypic R	Total phenotypic results
**Penicillin[a]**	24	22	2	98
**Levofloxacin**	3	0	3	74
**Clindamycin**	3	3	0	76
**Chloramphenicol**	1	1	0	97
**Erythromycin**	14	10	4	173
**Tetracycline**	5	2	3	98
**Co-trimoxazole**	13	11	2	122

[a] Out of 24 (and 22 WGS: R and Phenotypic:S) discordant results, 14 have novel PBPs.

[a]Penicillin susceptibility was determined using meningitis cut off by CLSI

Table S6. Percentage of resistant isolates against commonly used antibiotics stratified by patients' age

Antibiotic	Patients' age			Total
	**Under or of 5 years**	**Over 5 years**	**p for difference**	
Co-trimoxazole	96.6% (112/116)	90.1% (64/71)	0.46904	94.2% (179)
Penicillin	60.3% (70/116)	56.3% (40/71)	1	58.9% (112)
Tetracycline	46.6% (54/116)	62% (44/71)	0.27471	53.2% (101)
Doxycycline	46.6% (54/116)	62% (44/71)	0.27471	53.2% (101)
Erythromycin	25% (29/116)	33.8% (24/71)	0.66528	29.5% (56)
Clindamycin	11.2% (13/116)	12.7% (9/71)	1	15.3% (25)

Cefuroxime	2.6% (3/116)	7% (5/71)	0.4986	4.2% (8)
Chloramphenicol	3.4% (4/116)	4.2% (3/71)	1	3.7% (7)
Meropenem	0.9% (1/116)	4.2% (3/71)	0.4986	2.1% (4)
Cefotaxime	0.9% (1/116)	1.4% (1/71)	1	1.1% (2)
Ceftriaxone	0.9% (1/116)	1.4% (1/71)	1	1.1% (2)
Multidrug resistance	35.3% (41/116)	53.5% (38/71)	0.23982	43.1% (82)
